# Stronger proprioceptive BOLD-responses in the somatosensory cortices reflect worse sensorimotor function in adolescents with and without cerebral palsy

**DOI:** 10.1016/j.nicl.2021.102795

**Published:** 2021-08-21

**Authors:** Timo Nurmi, Julia Jaatela, Jaakko Vallinoja, Helena Mäenpää, Harri Piitulainen

**Affiliations:** a Faculty of Sport and Health Sciences, University of Jyväskylä, FI-40014 Jyväskylä, Finland; b Department of Neuroscience and Biomedical Engineering, Aalto University, FI-02150 Espoo, Finland; c Aalto NeuroImaging, Aalto University, FI-02150 Espoo, Finland; d Pediatric Neurology, New Children’s Hospital, University of Helsinki and Helsinki University Hospital, FI-00029 Helsinki, Finland

**Keywords:** Passive movement, SM1, SII, Kinesthesia, Hemiplegia, Diplegia

## Abstract

Cerebral palsy (CP) is a motor disorder where the motor defects are partly due to impaired proprioception. We studied cortical proprioceptive responses and sensorimotor performance in adolescents with CP and their typically-developed (TD) peers. Passive joint movements were used to stimulate proprioceptors during functional magnetic resonance imaging (fMRI) session to quantify the proprioceptive responses whose associations to behavioral sensorimotor performance were also examined.

Twenty-three TD (15 females, age: mean ± standard deviation 14.2 ± 2.4 years) and 18 CP (12 females, age: mean ± standard deviation, 13.8 ± 2.3 years; 12 hemiplegic, 6 diplegic) participants were included in this study. Participants’ index fingers and ankles were separately stimulated at 3 Hz and 1 Hz respectively with pneumatic movement actuators. Regions-of-interest were used to quantify BOLD-responses from the primary sensorimotor (SM1) and secondary (SII) somatosensory cortices and were compared across the groups. Associations between responses strengths and sensorimotor performance measures were also examined.

Proprioceptive responses were stronger for the individuals with CP compared to their TD peers in SM1 (p < 0.001) and SII (p < 0.05) cortices contralateral to their more affected index finger. The ankle responses yielded no significant differences between the groups. The CP group had worse sensorimotor performance for hands and feet (p < 0.001). Stronger responses to finger stimulation in the dominant SM1 (p < 0.001) and both dominant and non-dominant SII (p < 0.01, p < 0.001) cortices were associated with the worse hand sensorimotor performance across all participants.

Worse hand function was associated with stronger cortical activation to the proprioceptive stimulation. This association was evident both in adolescents with CP and their typically-developed controls, thus it likely reflects both clinical factors and normal variation in the sensorimotor function. The specific mechanisms need to be clarified in future studies.

## Highlights

• Cerebral palsy adolescents had stronger proprioceptive BOLD-responses.
• Cerebral palsy adolescents had worse sensorimotor performance.
• Stronger BOLD-responses were associated with worse sensorimotor performance.

## Graphical abstract

**Figure:**
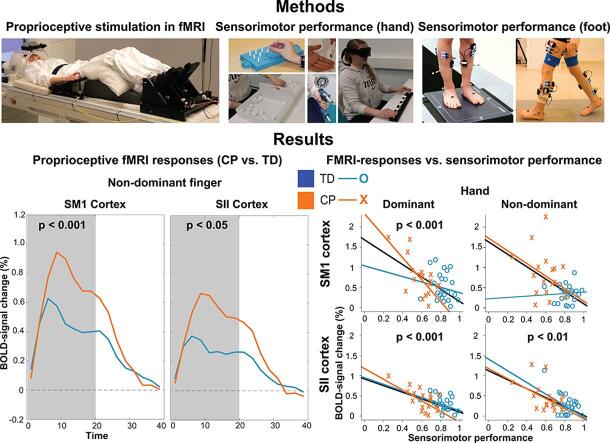

## 1 Introduction

Cerebral palsy (CP) is a group of permanent disorders in the development of movement and posture that are attributed to non-progressive disturbances in the developing brain (Rosenbaum et al., 2007; Mac Keith et al., 1958). The disorder is often accompanied by disturbances of sensation, perception, cognition, communication, and behavior in addition to co-occurring epilepsy and secondary musculoskeletal problems. The disorder has also relatively high prevalence of 2–2.5 out of 1000 births (Hagberg et al., 2001, Ashwal et al., 2004). Thus, better understanding of the neurophysiological effects of the disorder and the related specific neuronal mechanisms are important for enhancing the effectiveness and targeting of rehabilitation and treatments in CP. CP can be classified by topography, for instance, as hemiplegic (one side affected more) or diplegic (both sides are affected). Classification systems of motor function such as 5-point-scale Gross Motor Function Classification System (GMFCS; Palisano et al., 1997) are also widely used.

Smooth motor function depends largely on somatosensory afference from the body. More specifically, continuous somatosensory (primarily proprioceptive) feedback about the state of the locomotor system to the brain supports central nervous system motor circuits in controlling and adjusting on-going movements. Somatosensory afference has therefore a key role in motor learning and development of gross (*e.g.* gait) and fine (*e.g.* writing) motor skills (Metcalfe et al., 2005, Soechting and Flanders, 2008, Cascio, 2010). Proprioceptive afference provides information about the limb and body position, movement and forces (Proske and Gandevia, 2012). Proprioception is known to be impaired in CP (Goble et al., 2009, Wingert et al., 2009). This suggests that impaired proprioceptive afference has a role in the impaired motor function in CP.

Proprioceptive afference is processed widely in the neocortex, but majority of the fast direct thalamocortical cortical input is directed to the primary somatosensory (SI) cortex. In addition to the SI cortex, the secondary somatosensory (SII) cortex is important in processing the somatosensory afference. SI cortex seems to process early, lower-level stimulus features whereas SII cortex appears to integrate multimodal sensory information in a more bilateral manner (Inoue et al., 2002, Eickhoff et al., 2007). The primary motor (M1) cortex also receives direct, short-latency input from the proprioceptors and cortico-cortical input from the somatosensory cortices (Goldring and Ratcheson, 1972, Rosén and Asanuma, 1972, Asanuma and Arissian, 1984, Brovelli et al., 2004, Kocak et al., 2009). SI and M1 cortices are therefore often studied as a single functional unit, the primary sensorimotor (SM1) cortex and together with SII cortex form the most central part of the proprioceptive system when studying cortical proprioception.

Cortical proprioception can be studied using passive naturalistic movements of specific joints using different brain imaging methods (Müller-Putz et al., 2007, Sasaki et al., 2017, Sasaki et al., 2018, Alary et al., 2002, Druschky et al., 2003, Lange et al., 2001, Lolli et al., 2019, Nurmi et al., 2018, Piitulainen et al., 2015, Piitulainen et al., 2018, Piitulainen et al., 2021, Seiss et al., 2002, Vallinoja et al., 2021). For the continuous index finger movement, strongest BOLD-fMRI responses have been shown to be elicited by 3–6 Hz movements when using a blocked design (Nurmi et al., 2018). Cortical somatotopy and somatosensory processing are altered in CP patients evidenced by MEG and fMRI (Nevalainen et al., 2012, Papadelis et al., 2014, Piitulainen et al., 2018, Riquelme and Montoya, 2010, Riquelme et al., 2014). These cortical alterations appear to have functional significance, as they have been associated to worse motor performance in several motor disorders (Kusoffsky et al., 1982, Nociti et al., 2008).

Our primary aim was to clarify whether *proprioceptive responses in the sensorimotor cortices* of the fingers and ankles differ between adolescents with CP and their typically-developed (TD) peers. Secondly, we aimed to examine whether sensorimotor performance of the hand (motor skill) or foot (balance) were associated with the respective magnitudes of the cortical proprioceptive responses. Therapies and treatments in CP are most effective when started as early age as possible. For this reason, adolescents were chosen as participants. Our exploratory results will potentially offer basis for formulating new hypotheses in clinical research of CP.

## 2 Materials and methods

### 2.1 Participants

**Controls.** In total, 35 TD healthy controls were recruited for the study. Three of them quit prior or during the fMRI recording due to discomfort in the scanner or fear entering to it. In addition, nine participants were excluded from the fMRI analysis due to insufficient data quality such as absence of the activations and/or clear movement artifacts (2 participants), head movement exceeding 6 mm (5 participants), being on migraine medication during the measurement (1 participant) or having tic-symptoms (1 participant).

The remaining *23 healthy controls* (15 females, age: mean ± standard deviation, 14.2 ± 2.4 years) were included in the final analysis. Majority of the TD controls were right-handed (21 out of 23, Edinburgh Handedness Inventory mean score: 67.8; range: –87–100) and right-footed (20 out of 23, mean: 50.0; range: –60–100). The TD group was intellectually within normal variation (Wechsler Adult Intelligence Scale / Wechsler Intelligence Scale for Children mean score: 107.6, SD: 15.2; range: 77 –132; three participants missing a test score). See Table 1 for TD participant demographics.

**Table 1:** Participant demographics.

	Group / CP type	Dominant side (Hand)	Dominant side (Foot)	Sex	Age
1	TD	Right	Right	Male	13
2	TD	Right	Right	Male	12
3	TD	Right	Right	Female	12
4	TD	Right	Right	Female	15
5	TD	Right	Right	Female	16
6	TD	Right	Right	Female	12
7	TD	Right	Right	Female	12
8	TD	Right	Left	Male	17
9	TD	Right	Right	Female	17
10	TD	Left	Left	Male	17
11	TD	Right	Right	Female	17
12	TD	Right	Right	Male	13
13	TD	Right	Right	Female	16
14	TD	Right	Right	Male	13
15	TD	Right	Right	Female	14
16	TD	Right	Right	Female	14
17	TD	Right	Right	Male	11
18	TD	Left	Left	Female	12
19	TD	Right	Right	Female	10
20	TD	Right	Right	Male	12
21	TD	Right	Right	Female	10
22	TD	Right	Right	Female	16
23	TD	Right	Right	Female	16
24	CP/HP	Left	Left	Female	17
25	CP/HP	Left	Left	Female	10
26	CP/HP	Left	Left	Male	13
27	CP/HP	Left	Left	Female	11
28	CP/HP	Right	Right	Female	15
29	CP/HP	Left	Left	Male	10
30	CP/HP	Right	Right	Female	16
31	CP/HP	Left	Left	Female	15
32	CP/HP	Left	Left	Female	17
33	CP/HP	Right	Right	Female	13
34	CP/HP	Left	Left	Female	10
35	CP/HP	Left	Left	Female	14
36	CP/DP	Right	Right	Male	10
37	CP/DP	Left	Left	Male	12
38	CP/DP	Left	Left	Male	11
39	CP/DP	Right	Right	Female	15
40	CP/DP	Right	Right	Female	15
41	CP/DP	Right	Right	Male	15

Table shows participant demographics including group/CP type, dominant side of the hands and feet, sex and age.

**Patients.** In total 31 participants with CP (GMFCS classification of 1–2) were recruited to the study. Six of them quit due to discomfort in the scanner or fear entering to it, two were excluded due to absence of activations and/or clear movement artifacts, and five of them were excluded due to head movement exceeding 6 mm.

The remaining *18 participants* were included in the final analysis (12 hemiplegic and 6 diplegic participants; 12 females, age: mean ± standard deviation, 13.8 ± 2.3 years). Seven CP participants were right-handed (three hemiplegic and four diplegic) and 11 were left-handed (mean: –16.7, range: –100–100) and seven of them were right-footed (three hemiplegic and four diplegic; mean: –20.2; range: –100–89). For the hemiplegic participants, handedness and footedness were congruent with the clinical definition of their more affected side. The CP participants were cognitively mostly within normal variation (Wechsler Adult Intelligence Scale / Wechsler Intelligence Scale for Children mean score: 90, SD: 20.7, range: 43–117; five participants missing a test score).

The study was conducted according to declaration of Helsinki. All participants were 10–18 years of age. All participants gave their written consent, and in the case of minors, also their custodian gave a written consent. The protocol was approved by the ethics committee of Aalto University and Hospital District of Helsinki and Uusimaa (HUS). See Table 1 for detailed CP participant demographics and Table 2 for their lesion information.

**Table 2:** CP-participant lesion information.

	CP type	Lesion type	Side of lesion	Lesion location (MNI coordinates)	Lesion size (mm^3^)	MRI description
24	HP	infarction	Left	−54, −30, 57	17,573	Large lesion in left parietal lobe
25	HP	infarction	Left	−62, 8, 17	19,470	Large lesion in left middle frontal lobe
26	HP	infarction	Left	−63, −46, 31	67,544	Very large lesion covering left parietal and parts of superior temporal and posterior frontal lobes
27	HP	PVL	Left/Right	−14, −16, 26 17, 23, 13	1826 + 444	slightly enlarged posterior left lateral ventricle; small lesion in front of right anterior lateral ventricle
28	HP	PVH/infarction, HIBI, PVL	Right	14, 2, 23	1295	slightly enlarged posterior right ventricle
29	HP	lHI, IVH, WMI, PVL, WD	Left	−18, −5, 42	37,054	extensively enlarged left ventricle
30	HP	infarction	Right	10, −5, 22	6654	Slight enlargement of right lateral ventricle
31	HP	unknown	Left	−15, −20, 25	2764	Slight enlarged left lateral ventricle
32	HP	lHI, mild PVL	Left	−16, –23, 25	1006	slightly enlarged left lateral ventricle
33	HP	infarction	Right	40, −10, 17	65,833	Very large lesion in anterior-posterior-axel in the lateral regions covering frontal, temporal and parietal regions and making contact with an enlarged ventricle
34	HP	infarction	Left	−11, −11, 22	18,613	large enlargement of left venticle
35	HP	perinatal injury	Left	−26, −58, 3	675	slightly englarged ventricle
36	DP	PVL, local ischemia	None	(no visible lesion)	0	no visible lesion
37	DP	HIBI, PVL	Left	−24, −67, 4	381	Very slight enlargement of left inferior lateral ventricle
38	DP	PVL	None	(no visible lesion)	0	no visible lesion
39	DP	unknown, normal MRI	None	(no visible lesion)	0	no visible lesion
40	DP	unknown	Right	15, −10, 25	2566	Slight enlargement of right lateral ventricle
41	DP	unknown	Right	28, 24, 53	336	Very small lesion in right anterior frontal lobe

CP participants’ lesion information (type, side, location, size, MRI description). Lesion type was diagnosed by a clinician whereas MRI description was evaluated by a researcher (author of this article). Abbreviations: PVL, periventricular leukomalacia; PVH, periventricular hemorhage; HIBI, Hypoxic-ischemic brain injury; lHI, local hypoxia–ischemia; WMI, white matter injury; WD Wallerian degeneration.

### 2.2 Handedness and footedness

The hand and foot dominance were defined according to the Edinburgh Handedness Inventory (Oldfield 1971) and Waterloo Footedness Questionnaire Revised (Elias et al., 1998) test scores for healthy controls and diplegia patients. For the hemiplegia participants, the contralesional side was always defined as the dominant side (i.e. the functionally less affected side; the less affected side was always also the dominant side according to the handedness and footedness tests).

### 2.3 MRI equipment and parameters

Structural and functional imaging were carried out using a 3 T-MAGNETOM Skyra whole-body scanner (Siemens Healthcare, Erlangen, Germany). A 32-channel head coil was used. The measurements were carried out in Advanced Magnetic Imaging (AMI) Centre of Aalto Neuroimaging infrastructure in Aalto University, Espoo, Finland.

An anatomical T1 (MPRAGE) was obtained with 176 slices with slice thickness of 1 mm without an interslice gap and a 256x256 matrix with a field-of-view (FOV) of 256x256 mm, yielding a voxel size of 1x1x1 mm. Orientation of the structural image was sagittal. Repetition time (TR) was 2.53 s and echo time (TE) was 3.3 ms. Flip angle was 7°.

The functional images were obtained using a standard Echo-Planar Imaging (EPI) spin-echo sequence with a TR of 2.5 s and a TE of 30 ms. The functional volumes consisted of 44 slices with slice thickness of 3 mm without an interslice gap whose FOV was 192x192 mm with a 64x64 matrix yielding a voxel size of 3x3x3 mm. Flip angle was 90°.

### 2.4 Proprioceptive stimulation in fMRI

**Fig. 1:**
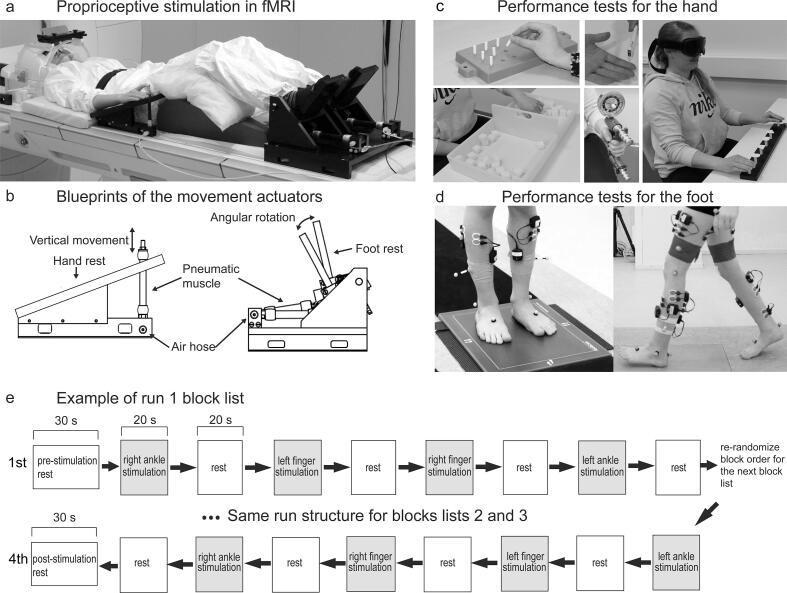
Measurements and equipment. (a) Experiment setup in fMRI. The participant lays on the MRI-table with the movement actuators attached to the index-fingers and feet. (b) Blueprints of the index finger and ankle movement actuators. (c) Sensorimotor performance tests for the hand, including 9-hole-peg, Box-and-Block, tactile monofilament, Jamar-strength and shape recognition tests. (d) Sensorimotor performance tests for the foot, including standing postural-balance and gait-variability test. (e) Example structure of run 1 with first and last block lists shown. Run 2 has a similar structure with more block lists. Note that the block order is different for each participant.

Four custom-made pneumatic movement actuators were used to evoke flexion–extension movements of the right and left index finger (for a detailed description of the actuators, see Nurmi et al., 2018, Piitulainen et al., 2015) and sagittal plane rotations of right and left ankle joints. These proprioceptive stimulators consisted of a plastic frame and an artificial pneumatic muscle (Fig. 1 a, b). The fingertips were attached to the pneumatic muscle with a surgical tape, and the feet were attached to the footrests using elastic straps. Surgical tape was also wrapped around participants’ index fingers to minimize tactile activation during stimulation. Participants’ hands and distal part of the arms rested on the upper plate of the finger-movement-actuator.

The artificial muscle moved along its longitudinal axis when its internal air pressure (1–5 bar) changed. The pressure was regulated by a solenoid valve (SY5220-6LOU-01F-Q, SMC Corporation, Tokyo, Japan) that was controlled by computer-generated trigger pulses. The solenoid valve was placed outside the MRI room and a 3.5-m non-elastic tube (internal diameter 2.5 mm) conveyed the airflow to the artificial muscle. The artificial muscle shortened in response to the increasing air pressure (opening of the valve), thereby flexing the finger or dorsiflexing the ankle joint, and then returned back to the initial position when the air pressure was released (closing of the valve).

For the index fingers, continuous 3-Hz movement was used. This movement frequency was found to be efficient in eliciting BOLD-responses in the somatosensory cortices (Nurmi et al., 2018). Ankle joint rotations were evoked at lower 1-Hz frequency, which lies well within the range of physiological foot actions and produces negligible head movement.

The stimulus runs with passive movement consisted of 20-s stimulation blocks with movement of one limb at a time, alternating with 20-s rest blocks without movement. Thus, the experiment consisted of four different experimental conditions: movement of (1) right index finger, (2) left index finger, (3) right ankle and (4) left ankle.

The stimulus runs with passive movement consisted of block lists (see Fig. 1e for an example of the first run; each row represents a block list in the figure). These block lists were created in order to present different conditions in semi-random order while still ensuring that the same condition is not presented several times in a row but different conditions are presented with similar incidence in every part of the stimulus run. A block list was defined to consist of alternating stimulation and rest blocks. Each of the experimental conditions in a stimulation block was presented in random order exactly once per a block list. When a block list is finished, another block list follows where the order of the presented stimulation blocks was again re-randomized. The first run consisted of four block lists and the second run consisted of eight block lists yielding 12x20-s stimulus blocks per condition in total, i.e., 4 min of stimulation per condition.

### 2.5 MRI protocol

During the (f)MRI scan, participants were laying on the MRI table with their limbs attached to the movement actuators (Fig. 1a). A pillow was placed under the participants’ calf and hamstrings. Two pillows were placed under the participant’s triceps, shoulders and elbows to give support for the arms. Respiration belt and pulse oximeter were attached to participant’s chest and middle or ring finger, and the respective signals were recorded throughout the fMRI scans using BIOPAC MP150 system (BIOPAC Systems Inc., Goleta, USA).

The MRI session consisted of four parts. First, a one-minute head-localizer scan was performed followed by a six-minute T1 anatomical scan. Then, 11 min long stimulus run was conducted. Second stimulus run lasted 22 min. The whole MRI session lasted 45–50 min. During the stimulus runs, a video of slowly changing pictures was shown to help the participant to remain alert.

### 2.6 Sensorimotor performance

**Hand.**Fig. 1c illustrates the sensorimotor tests. (1) A two-point discrimination test (range: 2–15 mm apart) to measure tactile discrimination ability of the hands in millimeters. Each digit in both hands was tested separately, and the results were averaged across the segments in both hands. (2) Semmes–Weinstein monofilaments test (Smith and Nephew Rolyan Inc., German-town, WI, USA) to evaluate tactile sensitivity thresholds using 2.8, 3.6, 4.3 and 4.6 mm thick monofilaments for 26 hand segments for both hands separately. (3) Jamar Hydraulic-Hand Dynamometer (Lafayette Instrument Company, USA) to measure unilateral hand-grip strength in kilograms. Average of three trials was used. (4) Box and block test (by Jean Hyres and Patricia Buhler, 1957) to quantify unilateral gross-manual dexterity as number of blocks moved from one box to another in 60 s. (5) Nine-Hole Peg test (see Mathiowetz et al., 1985) to quantify the unilateral fine-manual dexterity as the time (s) to place 9 pegs into 9 holes and back to the container. (6) Stereognosis test from the Sensory Integration and Praxis Test (SIPT; WPS, Torrance, California, US) test pattern to quantify unilateral shape recognition perception test of the hand measured by the number of shapes recognized correctly and the mean time (s) it took to recognize those shapes (two separate variables). (7) Bimanual bottle-opening test to quantify the ability to perform a bimanual task classified into four different performance levels based on the degree of involvement by the affected hand. (8) Unilateral kinesthetic accuracy test from the SIPT where the participant had to repeat finger position from initial position to end position on table surface measured as the distance. The movement was first guided by researcher and was then repeated by the participant. Five different directions and distances for each hand were performed and error was calculated in centimeters (average of all trials was used).

**Foot.**Fig. 1d illustrates tests for the lower limb (foot) bilateral sensorimotor performance. Standing postural stability was quantified using four standing balance tasks. The participant stood as still as possible on a platform recording plantar pressure distribution (0.5 m Hi-End Footscan® system, RSscan international, Brussels, Belgium) for 30 s per task. The four tasks were: (1) standing eyes open and feet shoulder width apart, (2) standing eyes closed and feet shoulder width apart, (3) standing with eyes open and feet together and (4) standing with eyes closed and feet together. Unit of measurement was center-of-force velocity (mm/s). Dynamic stability (standard deviation of step duration) was assessed using inertial measurement unit (NGIMU, x-io Technologies Limited, Bristol, UK) recordings during three walking tasks (for details, see Piitulainen et al., 2020a) (5) normal walking, (6) carrying a tray with a cup on it and (7) listing words in a given category during walking. Mean standard deviation in step duration was used (s).

**Sum variables of sensorimotor performance.** Sum variables were constructed for hand and foot sensorimotor performance separately. Each measure was first normalized across the whole studied population to scale from 0 (value of the worst performance) to 1 (value of the best performance). Then, the test values were averaged for each participant to obtain the sum variable. In case of unilateral tests, the left and right hand/foot measures were pooled together. The internal consistency of the sum variables items were estimated using Cronbach’s alphas and ensuring the values were 0.7 or above, indicating acceptable internal consistency (Cronbach, 1951, Nunnally, 1978).

**Comparison of sensorimotor performance between groups**. All of the single sensorimotor test values were compared between the CP and TD groups using MANOVA (hand and foot tests were tested separately) and post-hoc tests. Note that no imputation was used for these values.

Likewise, the overall sensorimotor performance of hands and feet were compared between the CP and TD groups using MANOVA (hand and foot tests were tested separately) and post-hoc tests. Imputation was used for these the overall sensorimotor performance values.

### 2.7 MRI preprocessing

The (f)MRI data of each participant was preprocessed using Matlab (R2016b, Mathworks, Natick, Massachusetts, United States) with a custom script using SPM12 functions (Wellcome Department of Imaging Neuroscience, University College London, UK). At first, both the structural and functional volumes were converted from Dicom to Nifti format.

The functional volumes were slice-time-corrected, motion-corrected by realigning to the last functional volume, functional volumes co-registered to anatomical volume. A tool called Drifter (Särkkä et al., 2012) was used to remove respiration and pulse artefacts using the physiological signals (pulse and respiration) recorded. Then, the data was smoothed with a kernel of 6 mm with the functions in SPM. A temporal high-pass filters of 334 s and 658 s were applied to each of the runs respectively in order to compensate for the signal drift.

Next, the timings and durations of the stimulation blocks were extracted, and a design matrix was constructed accordingly. The design matrix contained also six movement regressors that were used to reduce the confounding effect of movement artefacts. A general linear model (GLM) analysis was applied to obtain the beta-values of each voxel in response to the different stimulus blocks. This was done by having a canonical haemodynamic response function convolved with the stimulus columns of the design matrix. Finally, contrast images of each participant were constructed.

### 2.8 FMRI analysis

All analysis steps (time-courses, responses strength analyses) were performed in participants’ native space except when region of interest (ROI) locations were compared in the common MNI space and were projected into MNI152 template for visualization purposes.

**ROI construction and response time-courses.** Functional ROIs were constructed from the contrast images using the Marsbar toolbox (MARSeille Boîte À Région d’Intérêt; Marseille, France; version: 0.44). First, for the finger, SM1 cortex ROI was defined as activation posterior to the hand knob of the precentral M1. For the ankle, SM1 cortex ROI was defined as activation in the postcentral mesial wall of paracentral lobule (foot area). The SII cortex ROI was defined as activations within an/or proximity to the posterior insula for contra- and ipsilateral SII cortex separately. In case of the participant having more anterior activation only at M1 cortex, more anterior region, likely covering parts of M1 was used. In case of the CP group participant having lesion near or at the anatomical region of SM1 or SII cortices, primary activation in proximity of these regions was selected.

Next, a sphere of contralateral SM1 or SII cortex and 6 mm (leading to a ROI size of approximately 900 mm^3^ with slight variation between the participants due to discrete spatial data) radius was constructed. This sphere was positioned around the main activation center. After the ROIs were constructed, Marsbar functions were used in a custom Matlab script to extract time-courses for percent-of-signal-change. The value at each time-point was estimated using Marsbar’s finite impulse response model. Then, the value at the peak-response time-point was extracted. Time-courses correspond the average value across all voxels within a given ROI.

After the individual time-courses were constructed, a group-level mean time-course was computed by averaging the individual-level time-courses separately for TD and CP groups. The 95% confidence interval was also calculated for the group-level time-courses.

**BOLD response strength.** The response strength was quantified using percent-of-signal change of the BOLD signal. Two Multivariate Analysis of Covariance (MANCOVA; R package: jmv-1.2.5) tests were used for the index fingers and ankles separately to test whether group (CP or TD; factorial variable), hand performance or foot performance (covariates) had a main effect on any of the BOLD-responses in the somatosensory cortices. These tests were false discovery rate (FDR)-corrected according to the number of tests in a test family involving BOLD-responses (i.e. 2). In addition, we controlled the effect of total cumulative head movement (i.e. the sum of head movement over each time point) during stimulation, handedness/footedness score and age as potentially confounding covariates and sex as potentially confounding factor. MANCOVA assumptions were tested (see supplementary material / MANCOVA assumptions for more details) and Pillai’s trace (Pillai, 1955) selected as a robust test statistic.

Equivalence testing (R package: equivUMP, function: equiv.test; Wellek, 2010) where the null hypothesis is reversed (i.e. null hypothesis: there is difference between index finger and ankle stimulation) was used to see if head movement during finger stimulation was similar to head movement during ankle stimulation.

Effect sizes of group differences (CP vs. TD) for the significant response strengths were calculated using Cohen’s d (Cohen, 1988). Correlations between hand or foot performance and the respective response strengths were also calculated. Missing values were imputed using expectation maximization (EM; Walczak and Massart, 2001; function imputeEM in R package: mvdalab, version 1.4).

**Relative contributions of individual sensorimotor performance tests.** In the case of significant main effect(s) of the overall sensorimotor performance (i.e. the sum variable for the hand or foot), we computed relative explained variance to univariate linear model of each sensorimotor performance test and Spearman’s correlation to the BOLD-response strength. This was done to tease apart the relative effects for each individual sensorimotor performance test used, which provide functionally and clinically relevant details about the CP population. To keep the number of statistical tests at minimum, no statistical tests were performed at this stage.

**ROI locations.** Participants’ individual ROI locations were transformed to common MNI coordinates to see how well they corresponded SM1 and SII cortex locations of a standard parcellation (AAL, v4) and for visualization purposes on a MNI152 template. First, original T1 images were co-registered to a Freesurfer (http://surfer.nmr.mgh.harvard.edu/; Dale et al., 1999, Fischl, 2012) T1 image of the participant (identical T1 images, different coordinate systems). This was done because we used a pipeline from other project that required Freesurfer image coordinate system using a custom Matlab scripting with SPM and Marsbar functions. Then, using FSL (Analysis Group, FMRIB, Oxford, UK; version: 6.0.3) commands, the obtained transformation matrix was used to transform the ROI centre-of-mass coordinates into the Freesurfer coordinate system. Linear affine transformation was applied from the T1 images to MNI152 (2 mm) in order to obtain a transformation matrix from the Freesurfer space into the MNI152 space. Next, the MNI152 transformation matrix was used to transform the ROI centre-of-mass coordinates into the common MNI152 space for visualization purposes. Based on the MNI152-ROI coordinates, an image was constructed that shows the ROIs on a MNI152 template. The coordinates were also fed into a labeling tool to see where each partcipants’ ROIs in MNI152 coordinates would be in the AAL atlas. This was done in order to see how well the MNI mappings corresponded to official parcellation of the brain in MNI coordinates.

**Lesion locations.** Lesion locations were determined by a semimanual procedure where lesion ROIs were first constructed by visually inspecting CP participants’ T1 MRI images. Then, voxels were manually marked as part of the lesion ROI when white or gray matter was missing using tools of the MRIcron software (version: 1.0.20190902). In the case of enlarged ventricles, the enlarged ventricle was compared to the ventricle in the other hemisphere and lesion ROI was manually determined to be approximately the size difference of the ventricles between the hemispehres. The size of the lesion ROI was used to report the size of the lesion in mm^3^. After the manual determination of the lesion ROI voxels, procedures identical to building the sensorimotor cortex ROIs and projecting them to the MNI space was used to obtain MNI coordinates of the ROIs.

## 3 Results

The fMRI measurements were successful for all but one CP participant due to strong spasticity of the non-dominant hand. This participant was excluded from the time-series data. The missing response strength value of the spastic hand of this participant was filled in by imputation to the response strength data of 18 CP patients and 23 TD participants.

If the participant did not perform each sensorimotor-performance test, the values were filled in by imputation (4–5 TD and 3–5 CP participants for hand tests, and 2–3 TD and 1 CP participant(s) for foot tests). Only two TD and one CP participant had all of their sensorimotor test scores imputed.

### 3.1 Proprioceptive BOLD responses

**Fig. 2:**
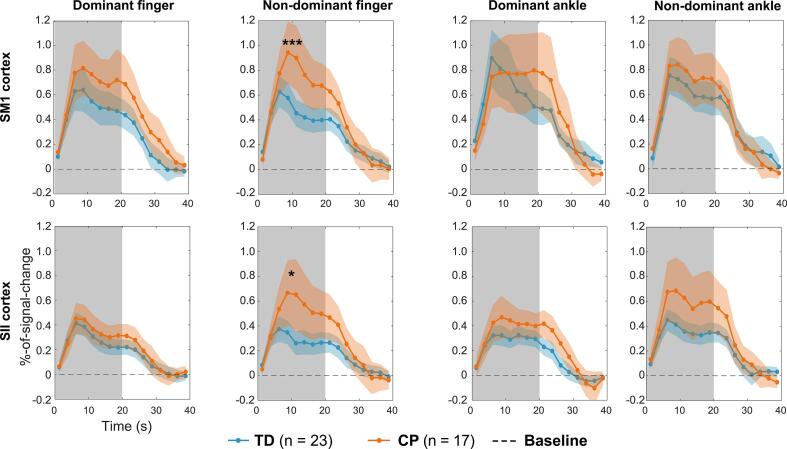
Group-average BOLD time-courses. Individual participants’ ROI time-courses (an average of the voxel time-courses of the ROIs) were average to yield group-average-time course. Percent-of-signal-change in BOLD-signal over time is used as the measure of the time-course. The grey background indicates the stimulation period. Confidence intervals of 95% are shown in colored shadings. Note that SI and SII cortices are always contralateral to the stimulated limb. * denotes *p* < 0.05 and ** denotes *p* < 0.01 in peak-BOLD-response amplitude between the groups (TD *n* = 23 and CP *n* = 18). Note the difference between the mean time-course sample size (CP group n = 17) and statistical test sample size (CP group n = 18) in CP as different sample sizes were used by using imputation for statistical testing.

Fig. 2 shows the group-average BOLD time-courses for the proprioceptive stimuli. The response strength (i.e. percent-of-signal change) reached its peak ~ 5–10 s after onset of the proprioceptive stimuli and returned to baseline at ~ 30–35 s.

The proprioceptive BOLD-response strengths of the index fingers differed between the groups (main effect: *p* < 0.05; FDR-corrected for tests related to fMRI-BOLD-resposes). Post-hoc tests detected stronger responses for the non-dominant (more affected side in CP) index finger both for SM1 (*p* < 0.001, *Cohen’s d*: 1.0) and SII (*p* < 0.05, *Cohen’s d*: 0.60) cortices. No group differences were significant for the ankles (main effect: p = 0.50).

### 3.2 Sensorimotor performance

Main between-group effect (CP vs*.* TD) was observed for both hand (p < 0.001) and foot (p < 0.01; FDR-corrected for behavioral only-related tests) sensorimotor tests. Table 5 presents the performance results and between group post-hoc test values for each test. The sensorimotor performance of CP group was significantly weaker compared to TD group in majority of the applied tests.

Main effect was also observed for the overall sensorimotor performance sum variables (p < 0.001). Post-hoc-tests revealed that group (CP vs. TD) had a main effect to hand (p < 0.001) and foot performance (p < 0.001). The TD group had better overall hand and foot performance indicated by the sum variables of the overall sensorimotor performance. Internal consistency of the sum variables was acceptable indicated by Cronbach’s alphas of 0.86 for hand and 0.87 for foot tests respectively.

### 3.3 Association between proprioceptive BOLD response strength and sensorimotor performance

**Fig. 3:**
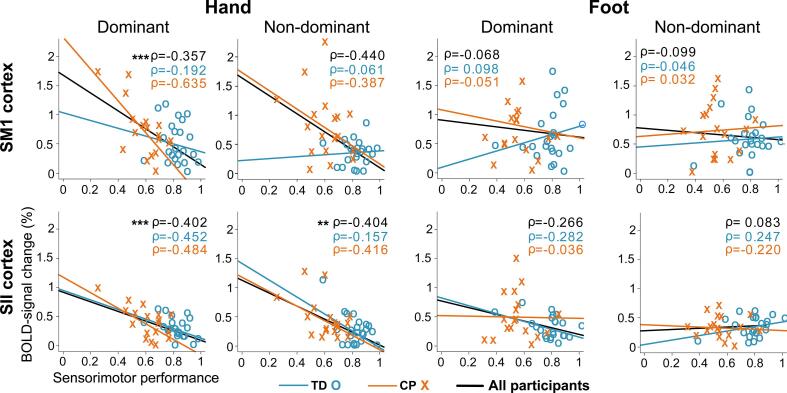
BOLD-responses versus overall sensorimotor performance. Associations between cortical response strength and sensorimotor performance in SI and SII cortices for hand and foot. Sensorimotor performance is the mean of each performance measure where each performance measure was normalized to a linear scale from 0 (lowest performance value among the participants) to 1 (highest performance value among the participants). * denotes p < 0.05, ** denotes p < 0.01 and *** denotes p < 0.001 for the whole examined population.

Fig. 3 shows Spearman correlation coefficients between the proprioceptive BOLD-response strength and sensorimotor performance. Significant negative correlations were observed only for the hand across the entire population (main effect *p* < 0.001). Post-hoc tests revealed that dominant hand function correlated significantly both with SM1 and SII cortex response strengths (p < 0.001), and non-dominant hand function correlated with the SII cortex response strength (p < 0.01), but not with SM1 cortex response strength (p = 0.11). No significant associations were detected for the foot performance and ankle responses. The negative correlations indicate that the worse sensorimotor performance of the hand was associated with stronger proprioceptive BOLD-response strength in the SM1 and SII cortices.

Table 6 shows how each individual sensorimotor performance test contribute to the BOLD response strength variance in each of the cortical ROI both in the case of the linear univariate model and Spearman correlation.

### 3.4 Lesion and ROI locations

Table 2 shows lesion locations in MNI coordinates in addition to lesion side, type and description.

**Finger ROI locations.** According to the AAL labels (see supplementary material/ AAL labels tables), 16/23 of TD and 13/18 of CP participants had their dominant SM1 ROI in the SI cortex (postcentral regions), 2 of both TD and CP participants in M1 cortex (precentral regions). Other regions included inferior parietal (1 TD participant). Four TD and 3 CP participants had their ROIs in undefined regions.

For the non-dominant SM1, 17/23 of TD and 9/17 (one participant had no ROI and response strength was imputed) of CP participants had their SM1 ROI in SI cortex, 5 of TD and 2 of CP participants had their SM1 ROI in M1. Other regions included superior frontal regions (2 CP participants). One TD and 4 CP participants had their ROIs in undefined regions.

For the SII cortex of the dominant finger, 8/23 of TD and 5/18 of the CP participants in the supramarginal regions, 6 of TD and 10 of the CP participants in the Rolandic opercular regions 5, of TD and 1 CP participants had their ROIs in the postcentral regions and 4 of TD and 2 of CP participants in the superior temporal regions.

For the non-dominant SII cortex, 17/23 TD and 4/17 (one participant had no ROI and response strength was imputed) CP participants had their ROIs in Rolandic opercular regions, 3 TD and 4 CP participants in supramarginal regions and 1 TD and 4 CP in postcentral regions. Other regions included superior temporal (1 TD and 1 CP participant), inferior temporal (1 CP participant), insula (1 CP participant), Hescl region (1 CP participant). One TD and CP participant had their ROIs in undefined regions.

**Ankle ROI locations.** The dominant SM1 ROI was located in the paracentral lobule in 11/23 of TD and 9/18 of CP participants and in precuneus in 5/23 of TD and in 4/18 of CP participants. Other regions included postcental regions (4 TD and 2 CP participants), superior parietal regions (2 TD participant) and supplementary motor regions (2 CP participants). One TD and 1 CP participant had their ROIs in undefined regions.

For the non-dominant SM1 ankle ROIs, 12/23 TD and 11/18 CP participants had their ROI in paracentral lobule and 9/23 TD and 4/18 had their ROI in the postcentral regions. Other regions included superior parietal regions (1 TD participant), superior motor area (1 TD participant), precuneus (2 CP participants) and precentral regions (1 CP participant).

The dominant SII ROIs were located in supramarginal gyrus in 10/23 of TD and 8/18 of CP participants, superior temporal regions in 5/23 of TD and 3/18 of CP participants and in Rolandic operculum in 4/23 of TD and 6/18 of CP participants. Four TD and 1 CP participant had their ROIs in undefined region.

The non-dominant SII ROIs were located in supramarginal gyrus in 7/23 of TD and 6/18 of CP participants and in Rolandic operculum in 10/23 of TD and 2/18 of the CP participants. Other regions included superior temporal (1 TD and 3 CP participants), middle temporal regions (2 CP participants) and insula (2 CP participants). Five TD participants and 3 CP participants had their ROIs in undefined regions.

**Fig. 4:**
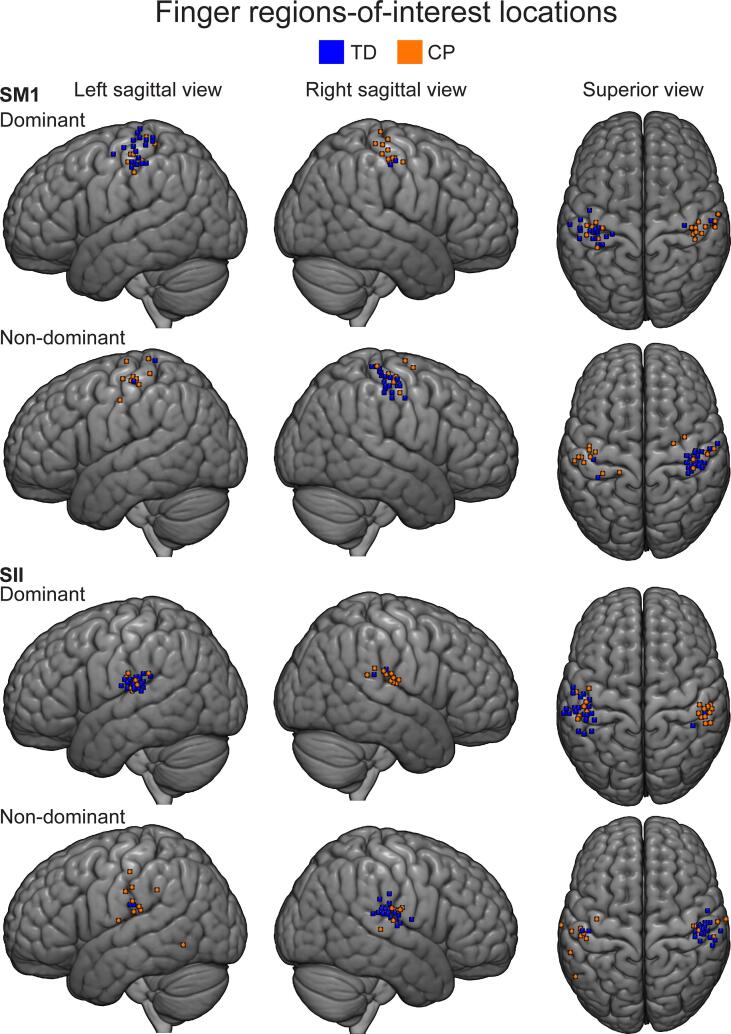
Finger peak locations projected on group brain. Participants’ finger peak locations of the ROIs are projected on a MNI152 template. Brain is shown from left-lateral, right-lateral and superior views. Single coordinate marked by a small cube indicating the centre-of-mass of the ROI is used.

**Fig. 5:**
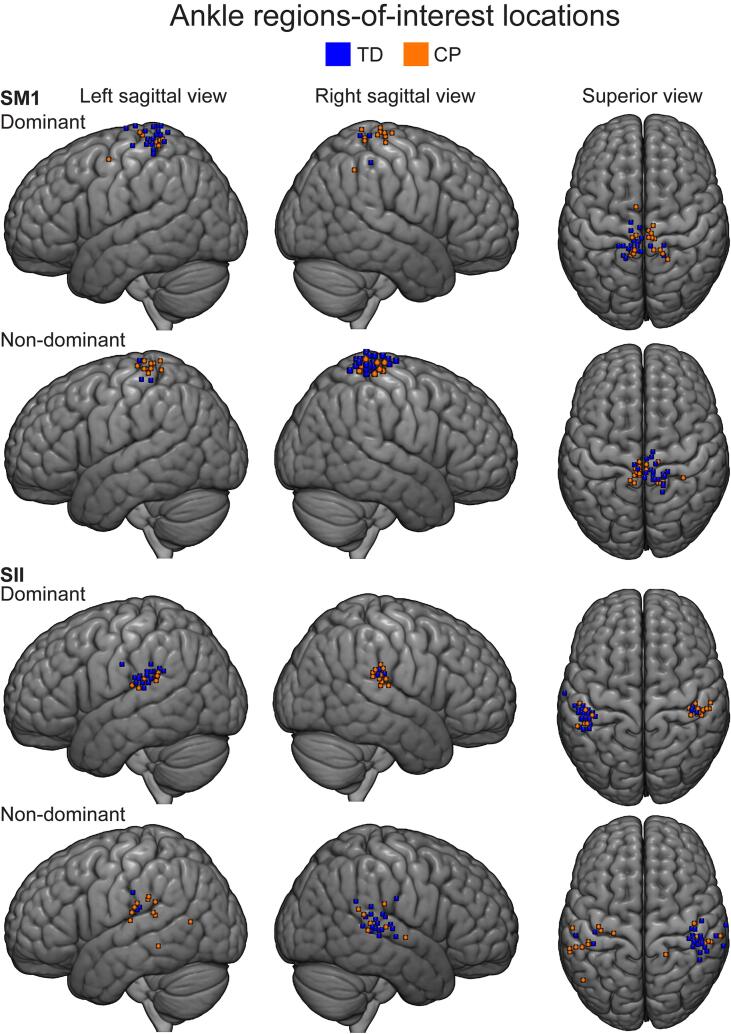
Ankle peak locations projected on group brain. Participants’ ankle peak locations of the ROIs are projected on a MNI152 template. Brain is shown from left-lateral, right-lateral and superior views. Single coordinate marked by a small cube indicating the centre-of-mass of the ROI is used.

**ROI information and visualization**. Fig. 4, Fig. 5 show finger and ankle ROI locations respectively, projected on a MNI152 template. Table 3, Table 4 show finger and ankle ROI sizes and locations in MNI space. AAL labels were also obtained and can be seen in supplementary material Table 1, Table 2 (supplementary_material_AAL_labels.doxc).

**Table 3:** Index finger ROI locations.

	Dominant finger		Non-dominant finger	
	SM1	SII	SM1	SII
Participant	ROI location (MNI)	ROI location (MNI)	ROI location (MNI)	ROI location (MNI)
**TD group**				
**1**	−26–28 76	−57–22 15	48–25 61	48–19 20
2	−53–32 50	−48–35 23	36–20 49	52–22 24
3	−45–8 57	−49–14 16	43–25 52	47–18 20
4	−41–22 51	−60–20 20	50–17 60	64–22 32
5	−40–23 67	−54–29 19	46–28 64	50–24 20
**6**	**−52**–**16 62**	−54–29 18	55–15 56	47–20 14
7	−37–37 67	−53–27 22	62–10 45	43–26 18
**8**	**−53**–**23 63**	−41–26 20	38–22 54	43–23 22
9	−38–22 47	−57–28 13	37–35 69	59–29 28
**10**	34–21 48	42–33 21	**−38**–**38 74**	−46–22 24
**11**	**−42**–**26 73**	−52–18 18	42–29 65	47–17 20
12	−40–32 68	−44–30 15	46–17 61	47–30 18
13	−42–24 59	−51–23 20	48–17 56	50–22 19
14	−35–37 69	−47–23 17	47–29 65	57–34 17
15	−39–25 50	−58–26 14	37–20 46	55–15 10
16	−38–32 64	−51–22 15	40–32 69	49–23 17
17	−38–26 54	−65–18 14	43–16 55	51–17 19
18	56–17 51	53–24 24	−42–23 58	−52–20 23
19	−38–28 69	−42–27 19	45–21 61	56–27 21
20	−61–20 51	−63–16 21	39–28 63	53–5 13
21	−37–24 50	−45–25 22	36–26 50	53–15 16
**22**	−31–30 51	−59–30 25	50–26 57	51–17 13
23	−52–23 51	−56–21 17	38–30 68	50–15 20
**CP group**				
24	43–26 63	52–23 22	−20–34 75	−39–12 11
25	48–27 57	54–18 16	None*	None*
**26**	59–22 51	57–16 15	**−40**–**17 73**	−57–60 −7
**27**	**62**–**11 50**	53–15 17	−52–21 60	−53–26 21
28	−34–23 44	−43–17 22	25–10 73	69–12 22
**29**	52–22 50	45–20 21	−45–12 44	**−69**–**17 33**
30	−47–29 52	−49–25 16	53–18 57	46–20 21
31	46–18 53	52–33 26	−44–21 59	−53–20 48
32	40–23 67	52–38 21	−34–35 64	−61–40 34
33	−43–22 57	−60–18 25	34–4 68	58–28 6
**34**	59–18 53	49–19 18	**−57**–**24 58**	−48–28 19
**35**	**42**–**28 73**	58–26 24	**−54**–**22 61**	−60–22 26
36	−38–39 65	−53–22 12	59–14 49	62–14 20
37	43–32 64	55–20 17	−45–15 60	−52–22 36
38	43–23 59	48–21 22	−50–26 56	−51–23 18
39	−47–21 57	−49–34 25	52–17 61	42–18 13
40	−40–19 51	−54–22 15	42–23 63	46, −20, 19
41	−38–32 69	−46–23 20	42–31 69	47, −21, 19

Table shows regions-of-interest of the index fingers center-of-mass locations in MNI coordinates. ROIs are spheres with radius of 6 mm and volume of approximately 900 mm^3^. Note that any participant whose MNI projection seemed to be outside cortex is bolded. *We were unable to position the participant’s hand adequately to the movement actuator, values imputed for response strength of the non-dominant hand.

**Table 4:** Ankle ROI locations.

	Dominant ankle		Non-dominant ankle	
	SM1	SII	SM1	SII
Participant	ROI location (MNI)	ROI location (MNI)	ROI location (MNI)	ROI location (MNI)
**TD Group**				
1	−6–35 68	−54–30 18	6–22 73	43–33 23
2	−2–31 76	−45–38 25	6–32 78	46, −29, 29
3	−6–37 60	−50–25 18	16–51 68	51–33, 18
4	−10–24 80	−47–30 19	10–40 70	44–26, 24
5	−18–37 70	−53–42 28	20–34 72	46–30, 22
6	−10–40 80	−45–43 27	8–39 67	40–28, 20
7	−6–40 73	−50–33 23	7–33 74	43–31, 24
8	17–36 51	41–27 26	−2–35 60	−56–22, 34
9	−8–32 66	−50–44 27	12–40 71	51–33, 24
10	6–37 71	42–25 23	−7–27 75	−44–25, 21
11	−12–40 76	−51–27 21	17–38 77	42–28, 22
12	−3–22 65	−46–36 21	7–17 75	**73**–**46, 32**
13	−8–42 73	−54–29 18	11–27 78	49–26, 28
14	−11–38 79	−49–30 14	16–39 82	69–28, 24
15	−21–37 62	−57–24 22	1–39 75	48, −30, 21
16	−11–40 73	−46–33 18	16–37 71	44–32, 22
17	−4–33 70	−45–27 22	2–34 67	54, −25, 21
18	16–44 71	45–30 22	−5–28 61	−38–26, 20
19	−11–17 78	−49–35 26	11–41 73	51, −28, 21
20	−6–41 80	−65–14 32	17–44 76	51, −34, 28
21	−5–41 65	−42–29 19	2–23 76	33, −24, 30
22	−17–45 68	−51–35 32	8–37 71	41, −30, 20
23	−15–48 75	−40–33 26	17–42 76	46–32, 24
**CP Group**				
24	9–40 68	50–28 20	−4–35 69	−60–21 17
25	16–43 76	53–27 29	−5–31 69	−62–41 −8
26	4–29 74	54–27 15	−10–42 75	−51–65 10
27	20–48 46	46–26 27	−6–30 74	−46–33 27
28	−6–4 55	−54–41 26	3–31 75	43–17 19
29	14–40 69	41–27 22	−6–33 65	−25–25 23
30	−7–41 65	−45–39 20	3–28 68	54–33 20
31	5–25 66	49–32 24	−4–26 71	−57–37 17
32	9–31 72	56–21 14	−8–37 73	−62–38 15
33	−7–28 75	−48–27 14	12–26 74	19–43 25
34	8–20 73	43–23 19	−8–43 68	−38–20 11
35	−4–36 73	−52–38 25	7–29 77	40–32 21
36	−8–29 73	−53–22 16	33–39 76	51–34 27
37	5–25 70	38–26 17	−3–29 69	−47–34 29
38	6–26 73	45–31 25	−2–33 66	−36–23 21
39	−9–42 69	−42–31 21	16–42 72	44–28 23
40	−10–40 68	−49–39 23	13–43 72	52–32 24
41	−10–44 70	−46–39 24	14–43 69	65–27 27

Table shows regions-of-interest of the ankle center-of-mass locations in MNI coordinates and sizes in mm^3^. ROIs are spheres with radius of 6 mm and volume of approximately 900 mm^3^. Note that any participant whose MNI projection seemed to be outside cortex is bolded.

**Table 5:** Sensorimotor tests.

	TD (n = 23)	CP (n = 18)	HP (n = 12)	DP (n = 6)	MANOVA (CP vs. TD)	Effect size (Cohen’s d)
**Hand tests**						
Box and Block (blocks/min)	72.2 ± 7.0	53.1 ± 12.1	51.3 ± 11.0	56.9 ± 14.6	**p < 0.001**	–1.4
Stereognosis (correct answers)	9.6 ± 0.7	8.3 ± 2.1	7.9 ± 2.4	9.1 ± 0.6	p = 0.09	–0.8
Stereognosis (s)	6.2 ± 2.6	12.6 ± 8.4	14.5 ± 9.9	8.8 ± 1.9	**p < 0.05**	1.0
Nine–Hole Peg (s)	17.7 ± 1.8	31.3 ± 18.8	37.3 ± 20.4	19.3 ± 5.6	**p < 0.001**	1.0
2–point discrimination(accuracy mm)	2.4 ± 0.3	2.7 ± 0.6	2.8 ± 0.8	2.5 ± 0.3	p = 0.58	0.6
Monofilaments (thicknes mm)	1.2 ± 0.1	1.2 ± 0.4	1.3 ± 0.4	1.1 ± 0.2	p = 0.73	0.3
Kinesthesia (accuracy cm)	2.3 ± 0.5	2.8 ± 0.6	2.7 ± 0.6	2.8 ± 0.6	p = 0.13	0.9
Grip strength (kg)	23.2 ± 6.4	16.7 ± 5.3	16.2 ± 4.0	17.6 ± 6.9	**p < 0.05**	–1.0
Bottle opening (level)	1.0 ± 0.0	1.8 ± 1.0	1.8 ± 0.8	1.6 ± 1.3	**p < 0.05**	1.1
**Foot tests**						
*Standing postural stability*						
Standing eyes open (mm/s)	6.6 ± 3.2	9.6 ± 3.1	9.0 ± 2.7	11.0 ± 3.7	**p < 0.05**	0.9
Standing eyes closed (mm/s)	7.5 ± 2.8	10.4 ± 3.2	9.4 ± 2.3	12.8 ± 4.0	**p < 0.05**	0.9
Feet together eyes open (mm/s)	8.6 ± 3.2	12.3 ± 3.1	12.6 ± 3.5	11.5 ± 2.0	**p < 0.01**	1.0
Feet together eyes closed (mm/s)	12.5 ± 4.1	17.2 ± 8.8	18.2 ± 9.8	14.7 ± 6.1	p = 0.06	0.7
*Dynamic gait stability*						
Normal gait	0.04 ± 0.02	0.07 ± 0.03	0.08 ± 0.03	0.06 ± 0.02	**p < 0.001**	1.2
Motor task constrained gait	0.04 ± 0.02	0.07 ± 0.02	0.07 ± 0.03	0.06 ± 0.02	**p < 0.01**	1.1
Cognitive task constrained gait	0.04 ± 0.02	0.07 ± 0.03	0.08 ± 0.03	0.06 ± 0.03	**p < 0.01**	1.0

Group and CP subtype differences between single sensorimotor tests. Values are given as mean ± SD. MANOVA was performed between groups (CP vs TD). Post-hoc test significance values are shown for specific sensorimotor tests. Effect sizes are also shown for the between group difference for specific sensorimotor tests.

**Table 6:** Bold response variance explained by sensorimotor tests of the hand.

	SM1 cortex				SII cortex			
	Dom. hand		Non-dom. hand		Dom. hand		Non-dom. hand	
TD group	Variance expl. (%)	ρ	Variance expl. (%)	ρ	Variancexpl. (%)	ρ	Variance expl. (%)	ρ

Box and Block	5	–0.24	24	−0.06	14	–0.46	12	–0.28
Stereognosis (correct answers)	4	−0.05	3	**0.03**	10	–0.28	6	–0.05
Stereognosis (s)	11	–0.24	3	−0.06	12	–0.52	10	–0.25
Nine–Hole Peg	14	**0.04**	3	**0.11**	22	–0.02	24	–0.27
2–point discrimination	5	**0.07**	6	**0.21**	2	−0.04	2	**0.07**
Monofilaments	>1	**0.01**	14	**0.25**	14	–0.39	2	–0.02
Kinesthesia	18	**0.03**	4	−0.08	7	–0.13	2	–0.05
Grip strength	39	–0.37	35	–0.37	7	–0.40	7	–0.29
Bottle_opening	4	–0.05	8	–0.14	13	–0.19	36	–0.46
**CP group**								
Box and Block	10	–0.52	50	–0.60	8	–0.51	43	–0.60
Stereognosis (correct answers)	19	–0.70	3	–0.31	15	–0.52	3	–0.19
Stereognosis (s)	24	–0.65	5	–0.41	35	–0.78	7	–0.29
Nine–Hole Peg	2	**0.11**	9	–0.19	10	**0.4**	6	–0.18
2–point discrimination	19	–0.45	>1	–0.18	10	–0.49	>1	–0.16
Monofilaments	9	–0.22	5	–0.33	11	–0.47	7	–0.45
Kinesthesia	2	–0.17	8	**0.16**	2	–0.24	2	–0.05
Grip strength	11	–0.51	14	–0.45	7	–0.44	25	–0.49
Bottle opening	4	–0.07	5	–0.12	2	**0.02**	7	–0.06

The univariate linear model sums up to 100% of relative variance. **Spearman’s correlation coefficients (ρ) to BOLD–response strength. Positive correlation values are indicated as bold.

### 3.5 Confounding covariates

None of the confounding factor were significant. The association between BOLD-response strength and cumulative head movement, age or sex or handedness were not significant for the finger (p = 0.36–0.93) or ankle covariates (p = 0.21–0.94).

**Head movement equivalence.** Mean cumulative head movement was 49.5 mm for right hand, 47.3 mm for left hand, 49.0 mm for right ankle and 46.2 mm for left ankle stimulation. Equivalence test revealed that mean head movement did not differ significantly between the different conditions (p < 0.001 for all; null hypothesis reversed).

## 4 Discussion

The main observation was that adolescents with CP showed higher sensorimotor cortical responsiveness to proprioceptive stimulation of their more affected index finger compared to healthy controls. Moreover, stronger proprioceptive responses were predominantly associated with worse sensorimotor performance of the hands across both groups — with the exception of the SM1 cortex of the non-dominant finger whose association was not significant. Notably, these findings were not replicated in the ankle joint, and this was not explained by head movement during the fMRI scanning but may be related to the sensorimotor control and function of the hands and feet that differ fundamentally, i.e., for example the cortex is more directly involved in the fine motor control of the distal hand whereas spinal and subcortical control is more pronounced in the coarser control of the lower limbs. Altogether, it seems that stronger responsiveness of the sensorimotor cortices of the index finger is associated with worse sensorimotor function of the hand in general whether clinical or non-clinical in essence.

### 4.1 Responsiveness of the sensorimotor cortices to proprioceptive stimulation in cerebral palsy

It is challenging to conclude how well our findings are in-line with the research literature, as the available prior evidence is limited and highly divergent between the studies. We are aware of only a single study using passive movements of the fingers in fMRI directly comparing CP and TD participants. In contrast to our results, Van de Winckel et al. (2013) reported no evidence of difference in response strength of the SM1 activation between CP and TD groups using passive movements of the index finger by a movement actuator in fMRI. However, the same investigators (Van de Winckel et al., 2013) reported that *active* movements of the hand do produce stronger activation of the SM1 cortex in the CP group, which is in line with our current observations. When using MEG with partly overlapping population of the same participants as in the current study, Piitulainen et al. (2020b) found that evoked-MEG responses to passive finger movements were weaker in diplegic participants than in healthy participants in the more affected hemisphere, with no differences between the hemiplegic and TD participants. Differences in MEG and fMRI methods or stimulation protocols might predominantly explain these discrepancies, i.e. MEG and fMRI measuring different aspects of neural function or using repetitive passive movements in fMRI vs. using single movements in MEG.

In the cutaneous tactile domain, Wingert et al. (2010) demonstrated weaker BOLD-responses of the SM1 cortex to active tactile discrimination task in fMRI when diplegia patients were compared to healthy participants, but no difference between CP and TD children (Van de Winckel et al., 2013). These results are in contrast with our findings. In-line with our results, Riquelme and Montoya (2010) reported enhanced early evoked MEG potentials and excitability in the SI cortex to tactile stimulation in participants with CP compared to healthy peers. Furthermore, Maitre et al. (2012) reported stronger contralateral late responses of the more affected hand when compared to the less affected hand in patients with CP.

Peripheral electrical stimulation of the median and tibial nerves activate a mixture of tactile and proprioceptive afferents, and thus is adequately relevant comparison to our proprioceptive stimuli. In contrast to our study, Guo et al., 2012, Trevarrow et al., 2021 observed weaker somatosensory responses in patients with CP than healthy peers using MEG. Even though electric stimulation likely stimulates also proprioceptive afference, these discrepancies to our study are not necessarily contradicting. Electric stimulation and naturalistic passive movements might still activate the sensorimotor cortices differently. The aforementioned partly contradictory findings may arise from different (1) imaging methods (i.e. fMRI versus MEG), (2) lesions types, (3) CP types, (4) modality (eg. tactile vs proprioceptive vs electric stimulation), (5) stimulation protocol (eg. single vs. repetitive movements), and/or (6) difference in the attentional engagement of the task (i.e. active top-down versus passive bottom-up).

### 4.2 Sensorimotor performance and its associations to proprioceptive responses

As expected, the CP group had significantly worse hand and foot sensorimotor performance. Majority of the specific performance tests indicated large effect size between the CP and TD participants. CP participants showed worse performance in tasks relying largely on proprioception, such as gross and fine motor skill, stereognosis and balance, or muscle force production (grip-strength test). Thus, these tests appear sensitive enough to identify sensorimotor impairment in the context of CP. Moreover, the dynamic stability of the gait in CP in partly same participants was impaired when compared to controls (see Piitulainen et al., 2020a). However, tactile sensitivity (2–point discrimination and monofilament test) was not significantly affected in our adolescents with CP when compared to the TD peers. The intact cutaneous tactile sense was somewhat surprising, although we did expect more problems in the proprioception-based tasks. Our patients with CP expressed distinct proprioceptive or kinesthetic impairments although they were relatively well functioning, *e.g.*, were able to move without assistive devices. Furthermore, the hemiplegic and diplegic patients showed similar sensorimotor performance, but the hemiplegic patients showed more impaired gross and fine motor skills, albeit tested unilaterally.

The sensorimotor performance was associated with the strength of the proprioceptive responses. Stronger cortical responses to proprioceptive finger stimulation indicated worse sensorimotor performance of the hand, in both CP patients and their healthy controls, with the exception of the non-dominant SM1 of the finger. In contrast to this result, Harrach et al. (2020) reported that stronger response strength in the sensorimotor cortices during an active hand motor task in fMRI was associated with better sensorimotor performance in children with stroke. However, our results are in-line with the research literature on experts such as pianist (Jäncke et al., 2000, Krings et al., 2000) and race car drivers (Bernardi et al., 2013) who tend to show less activation of the sensorimotor cortices than their less-skilled peers when performing a motor task within their expertise. Moreover, research in healthy aging suggest that decline in motor performance due to aging is associated with stronger activation of the sensorimotor cortices (Heuninckx et al., 2008, Mattay et al., 2002, Naccarato et al., 2006, Piitulainen et al., 2018, Ward and Frackowiak, 2003), albeit diverging results have also been obtained (Hutchinson et al., 2002, Wu and Hallett, 2005, Riecker et al., 2006, Landelle et al., 2020). It is noteworthy, that in contrast to our study, most of these studies (excluding Piitulainen et al., 2018) have used active motor tasks simultaneous with functional imaging.

The association between worse sensorimotor performance and stronger cortical response strength supports our main observation that the CP participants had stronger sensorimotor cortical responses to the proprioceptive stimulation. This was observed in their more affected hemisphere, i.e. in the regions controlling the more affected hand. The same association was detected also in the less affected hemisphere of CP and also for the healthy peers. Thus, it seems unlikely that the between-group differences are due to a confound, *e.g.*, ROI selection bias or spasticity, but reflect physiologically valid effect.

Finally, the lack of association between the response strength and sensorimotor performance at the lower limb might partly be due to the homogeneity of the lower limb performance tests (postural and dynamic balance). Whereas the upper limb performance tests covered a wide variety of upper limb functionality. Thus, the aspects of lower limb function measured might not have covered comprehensively the aspects being coupled with the function of the somatosensory cortices. There is a lack of standardized lower limb test battery that would assess the lower limb function comprehensively, especially in the proprioceptive domain.

### 4.3 Lesion and ROI locations

Enlargement of the lateral vertices was the most common apparent anatomical finding in our CP participants. This was expected as ventricular enlargement is found to be a significant risk factor for CP (Pinto-Martin et al., 1995). In our participants with clearly enlarged ventricles, the cortical responses were mostly in the expected locations. The enlarged ventricles often altered the morphology in vicinity or within the SII cortex, which made ROI identification more challenging in such patients. However, we were able to define the ROI adequately for all CP patients.

The ROI locations appeared to be spatially more variable in participants with CP than in their healthy peers, both in native and common MNI spaces, and especially in the more affected hemisphere. For this reason, the ROI localization was typically more challenging in the CP patients. For example, one patient (participant 3) had a large lesion covering nearly the entire SII cortex, and therefore the ROI of the more affected finger was identified posterior and inferior to a typical SII cortex location. Another similar example was participant 31, whose SII cortex location was identified superior to typical SII cortex location. For the healthy participants, both SM1 and SII cortices of the fingers were typically located in the expected post-central sulcus and posterior insula respectively. However, in few healthy participants the ROI in the SM1 cortex was identified in a more anterior M1 cortex. This is plausible as proprioceptive afference is directed also directly to the M1 cortex (Goldring and Ratcheson, 1972). In the common MNI space, some SM1 cortex ROIs (2–4 cases) were slightly mislocated with 1–2 mm outside from the cortex, possibly because of the smaller head size in our child participants compared to the MNI template (MNI152) constructed from adult data. In the native space, all the ROIs were located within the cortex. It is important to note that all analyses regarding the main hypothesis were performed in native space. Therefore, a failure to correctly map participant into the common MNI space is insignificant, since it was used only for visualization purposes and to provide response locations in the common coordinate system. We were able to use the valid anatomical and functional (i.e. activation) criteria at the individual anatomy (i.e. in native space), however, the SM1 cortex activations in some of the participants’ were located near the skull surface but still within the gray matter of the SM1 cortex.

### 4.4 Limitations

As expected for a CP population, the lesion location and size varied substantially within our CP participants from indiscernible or slightly enlarged ventricles to large lesions covering almost the entire SM1 and SII cortices of the affected hemisphere(s). The anatomical and symptomatic heterogeneity in CP populations is a common challenge for convergent conclusions about the disorder. The heterogeneity and limited availability of the patients are likely reasons for diverging observations in the research literature regarding the cortical sensorimotor function in CP. The number of CP participants was low also in the current study, and thus we could not estimate how lesion type or developmental onset affects the cortical proprioceptive responses. Our focus was on the somatosensory cortices, in which the laterality shifts are rare compared to the M1 cortex in CP (Thickbroom et al., 2001). Typically, the laterality shifts of the somatosensory cortices are associated with severe motor impairments (Lemée et al., 2019), whereas our study was focused to the adolescents with spastic CP who were able to walk without assistive devices (GMFCS 1–2).

Differences in handedness and footedness can also be a concern when interpreting the between-group results. The CP group was predominantly left-handed and footed (i.e. their more affected side was right) whereas opposite was true for the healthy controls. The sensorimotor performance and BOLD-response strengths were grand-averaged according to the dominance. However, this was the only reasonable approach, because our primary aim was to examine the effect of the brain lesion on the cortical proprioceptive processing in the more affected hemisphere. Moreover, pooling the participants according to limb dominance for the association analysis between the proprioceptive responses and sensorimotor performance might be another potential concern. It is not clear whether the typically-developed, non-dominant side of the healthy participants is directly commensurable to the non-dominant lesion-side of the CP participants. However, we assumed that the non-dominant side of the healthy participants is the best analogue for the lesion-side of the participants with CP when comparing the groups.

We examined patients with spastic CP, which could potentially enhance the proprioceptive BOLD- response strength. The spastic activity could sensitize the muscle spindles via gamma neuron activity. However, the muscle spindles are extremely sensitive to even to tiny length changes (as low as 5 µm during vibration) of their parent muscle even in perfectly relaxed passive conditions (Brown et al., 1967). Therefore, it is unlikely that the mild spasticity in some individuals would have significantly influenced the current results. This view is also supported by our observation that the worse sensorimotor performance was associated with the stronger cortical proprioceptive responses regardless of the participant having CP or not. We further mitigate this potential concern by not including CP participants with severe spasticity in their limbs.

Seven participants were excluded from the data due to excessive head movements during fMRI scanning. Rejection due to excessive head motion is typical in fMRI studies in children and adolescents (Rajagopal et al., 2014, Vanderwal et al., 2015). We attempted to minimize the head motions by presenting the participants a video of slowly changing pictures during the scanning. It has been shown that presentation of video during the fMRI scanning effectively reduced head movements in children and adolescents (Vanderwal et al., 2015). Furthermore, a rather liberal movement rejection threshold (6 mm) was applied and imputation of data in some participants when applicable. This was unavoidable, but the reader should be aware of these potential confounds when making interpretations of this and other studies with populations with elevated risk of excessive head movements.

Functional localizer was not used to identify the ROIs. The use of anatomical ROIs was not possible due to cortical lesions in the CP participants, and thus they did not always follow the normal somatotopic organization. To avoid excessive head movements, the scanning duration was limited as short as possible. For these reasons, the same fMRI was used in the selection of the ROIs locations and extracting the mean BOLD-signal in them. This approach can potentially bias the results (for details, see Poldrack, 2007). However, since robust significant effect of the lesion on the cortical responses was observed with significant correlation with the behavioral sensorimotor performance, it can be assumed that the aforementioned potential confounds negligibly affected the results.

### 4.5 Possible neural basis and future prospects

Stronger cortical activation to proprioceptive stimulation in CP and its association to worse sensorimotor function may be explained by several different, although speculative and possibly overlapping models. ‘The compensation hypothesis’ explains the findings by the inefficiency in the neural processing caused by the lesion, which are compensated by recruiting additional neural populations. Furthermore, the ‘dedifferentiation hypothesis’ presumes that the neural signaling itself is inefficient, as a larger population of neurons is processing the information in a less optimal or specialized manner. This is in similar vein with the interpretation by Piitulainen et al., 2018 who came to the suggestion that more efficient neural proprioceptive processing is achieved by more specific, smaller population of sensorimotor cortex neurons in a well-functioning sensorimotor system compared to a less well-functioning sensorimotor system.

Altered inhibition-excitation balance in the sensorimotor cortices with impaired inhibitory processes might be the neuronal mechanism behind the dedifferentiation hypothesis. Stronger BOLD-responses have been shown to correlate positively with glutamaergic excitation and negatively with GABAergic inhibition (Muthukumaraswamy et al., 2009, Donahue et al., 2010, Bednařík et al., 2015, Kurcyus et al., 2018, Stagg et al., 2011, Just and Sonnay, 2017). In addition, Piitulainen et al. (2018) suggested the impaired excitation-inhibition balance and Riquelme and Montoya (2010) enhanced excitability in the somatosensory cortices in CP. Moreover, post-movement beta rebound amplitude in the SM1 cortex correlates positively with higher GABA levels in the SM1 cortex (Gaetz et al., 2011, Cheng et al., 2017), and is weaker in individuals with CP participants (Hoffman et al., 2019) and especially in their more affected side (Pihko et al., 2014). Animal models of CP show hindered GABAergic inhibition and increased responsiveness of the SI cortex (Coq et al., 2008) and post-mortem studies of neonates report loss of GABAergic neurons associated with the perinatal brain injury (Robinson et al., 2006, Stolp et al., 2019).

A complementary explanation to altered inhibition-excitation balance might be altered structural and functional connectivity. Diffusion tensor imaging studies have demonstrated that thalamocortical pathways to somatosensory cortices may be structurally deficient in CP (Hoon et al., 2002, Hoon et al., 2009, Papadelis et al., 2018, Tsao et al., 2015), whereas functional connectivity studies point to stronger and more expansive intracortical sensorimotor functional connectivity (Burton et al., 2009). The enhanced functional connectivity in CP may propagate the proprioceptive stimulus related activity to wider cortical neuronal network which might partly explain the observed stronger BOLD responses in line with the ‘dedifferentiation hypothesis’.

Other kinds of explanations are possible as well. Since the percent-of-signal change is a relative measure against some baseline. It is therefore possible that the CP group simply had lower baseline activity in the somatosensory cortices while having similar response strength to stimulation as the TD group, leading to a higher relative activation during stimulation. This, in turn, might be due to the somatosensory cortices of the CP group processing less neural information in its non-stimulated, default state when the fingers are relatively still.

Lastly, it is also possible that the neurovascular coupling is affected by the lesion in the affected hemisphere in the CP patients. However, it is unlikely that differences in neurovascular coupling in the TD group would also have an inverse relationship with sensorimotor performance and which would therefore point to similar conclusion as the group differences, i.e. that higher responsiveness is related to worse motor performance whether measured with the behavioral measures or seen as an effect between the dominant and non-dominant side in the CP group.

### 4.6 Conclusions

Individuals with spastic CP showed stronger BOLD-responses to proprioceptive stimulation of the index finger in their more affected (by the brain lesion) sensorimotor cortices compared to healthy peers. The possible neuronal mechanism may include (1) impaired efficiency of the cortical proprioceptive processing due to *altered inhibition-excitation balance* (2) and/or *compensatory recruitment* of additional neuronal resources to accomplish adequately the proprioceptive processing. Moreover, the stronger cortical proprioceptive responses were predominantly associated with worse sensorimotor performance of the hands across the studied CP and healthy population. Future studies on cortical proprioceptive processing in CP could attempt to test the aforementioned hypotheses potentially explaining the stronger proprioceptive responses in CP.

## Funding

This work was supported by the Academy of Finland (grants #296240, #304294, #307250 and #3327288 to HP) and ”Brain changes across the life-span” profiling funding to University of Jyväskylä (grant #311877).; Jane and Aatos Erkko Foundation and Emil Aaltonen Foundation (grant #602.274) to HP.

## CRediT authorship contribution statement

**Timo Nurmi:** Conceptualization, Data curation, Formal analysis, Investigation, Software, Visualization, Writing - original draft, Writing - review & editing. **Julia Jaatela:** Investigation, Formal analysis, Writing - original draft, Writing - review & editing. **Jaakko Vallinoja:** Investigation, Formal analysis, Writing - original draft, Writing - review & editing. **Helena Mäenpää:** Conceptualization, Funding acquisition, Project administration, Writing - original draft, Writing - review & editing. **Harri Piitulainen:** Conceptualization, Funding acquisition, Investigation, Project administration, Supervision, Writing - original draft, Writing - review & editing.

## Declaration of Competing Interest

The authors declare that they have no known competing financial interests or personal relationships that could have appeared to influence the work reported in this paper.
