# Supplementary material for: Stronger proprioceptive BOLD-responses in the somatosensory cortices reflect worse sensorimotor function in adolescents with and without cerebral palsy
Source: Neuroimage Clin. 2021 Aug 21;32:102795. doi: 10.1016/j.nicl.2021.102795 (PMC8411230; doi:10.1016/j.nicl.2021.102795)
Supplement: Supplementary data 1 [file mmc1.docx]

# MANCOVA assumptions.

## Univariate assumptions

Normality was tested using Shapiro-Wilks test (R package: ‘car’ function: ‘shapiro.test’). Outlier analysis was done by first visually inspecting the Q-Q plots and performing Rosner’s test with the number of suspected outliers + 1 (this was done in order to ensure that the test was performed to a single most extreme value even when there were no suspected outliers from visual inspection).

## Univariate assumption results

**Normality of finger/hand variables.** For the finger response strength and hand performance variables, all but the response strength of SII cortex of the non-dominant finger in both the TD (Shapiro-Wilks : p=0.002) and CP (p=0.003) groups were normally distributed (p-values 0.06-0.78).

**Normality of ankle/foot variables.** For the foot variables, all but SI cortex of the dominant ankles responses (p=0.0007) and foot performance (p=0.01) were normally distributed. See Figure 1 (finger/hand variables) and 2 (ankle/foot variables) for details.

**Outliers in finger/hand variables.** Results of the univariate outlier analysis revealed that the response strengths of SII cortex of the non-dominant finger had one outlier in the TD group and two outliers in the CP group. Moreover, the hand performance of the CP group had one outlier.

**Outliers in ankle/foot variables.** For the ankle and foot related variables, one outlier was detected for the response strength of the SI cortex of the dominant ankle in the CP group and one outlier in the lower limb performance of the TD group.


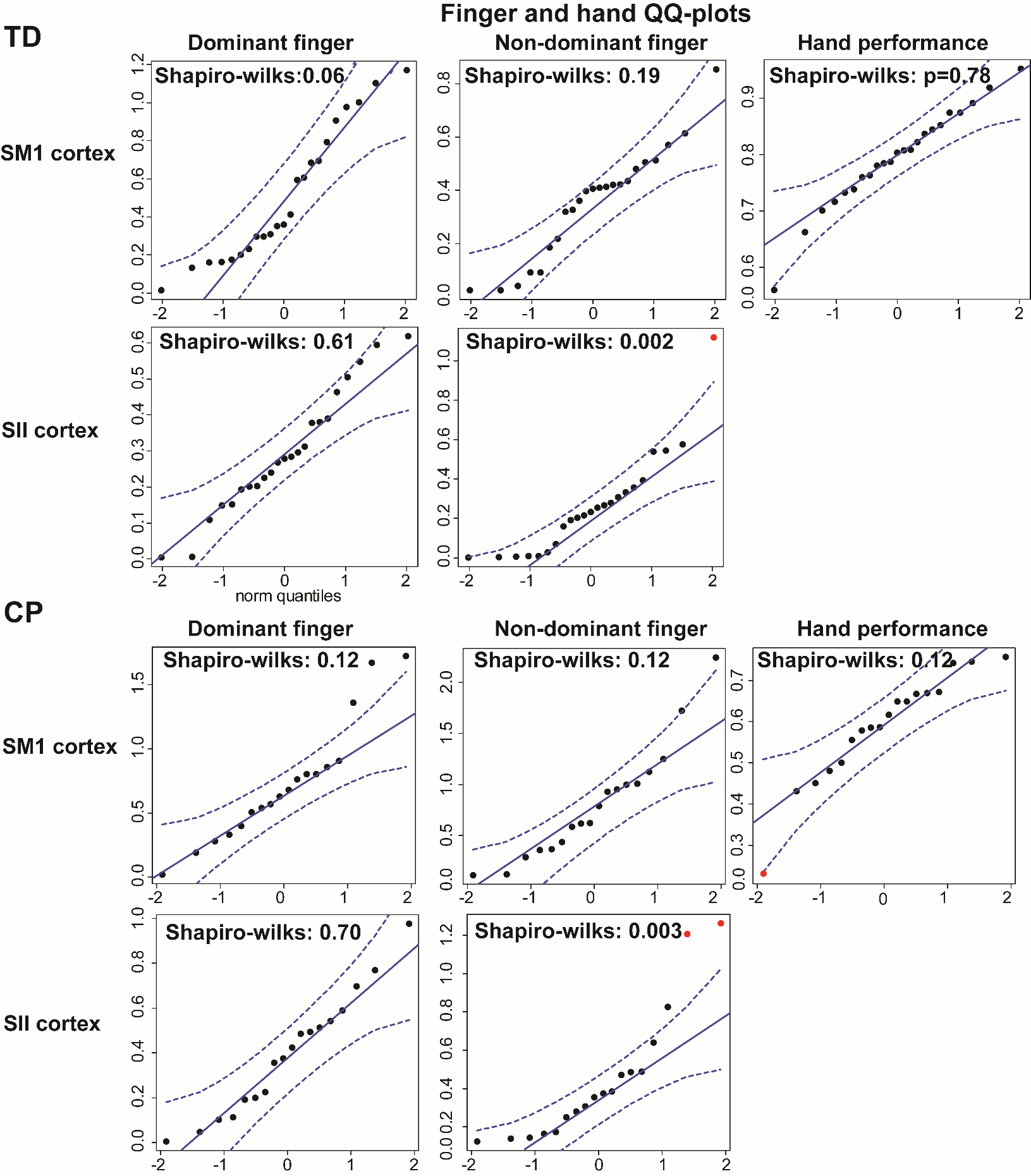


**Figure 1. QQ-plots of finger and hand related variables**. Figure 1 shows QQ-plots and Shapiro-Wilks test statistics for testing the normality of variables. Outliers are marked in red.

-


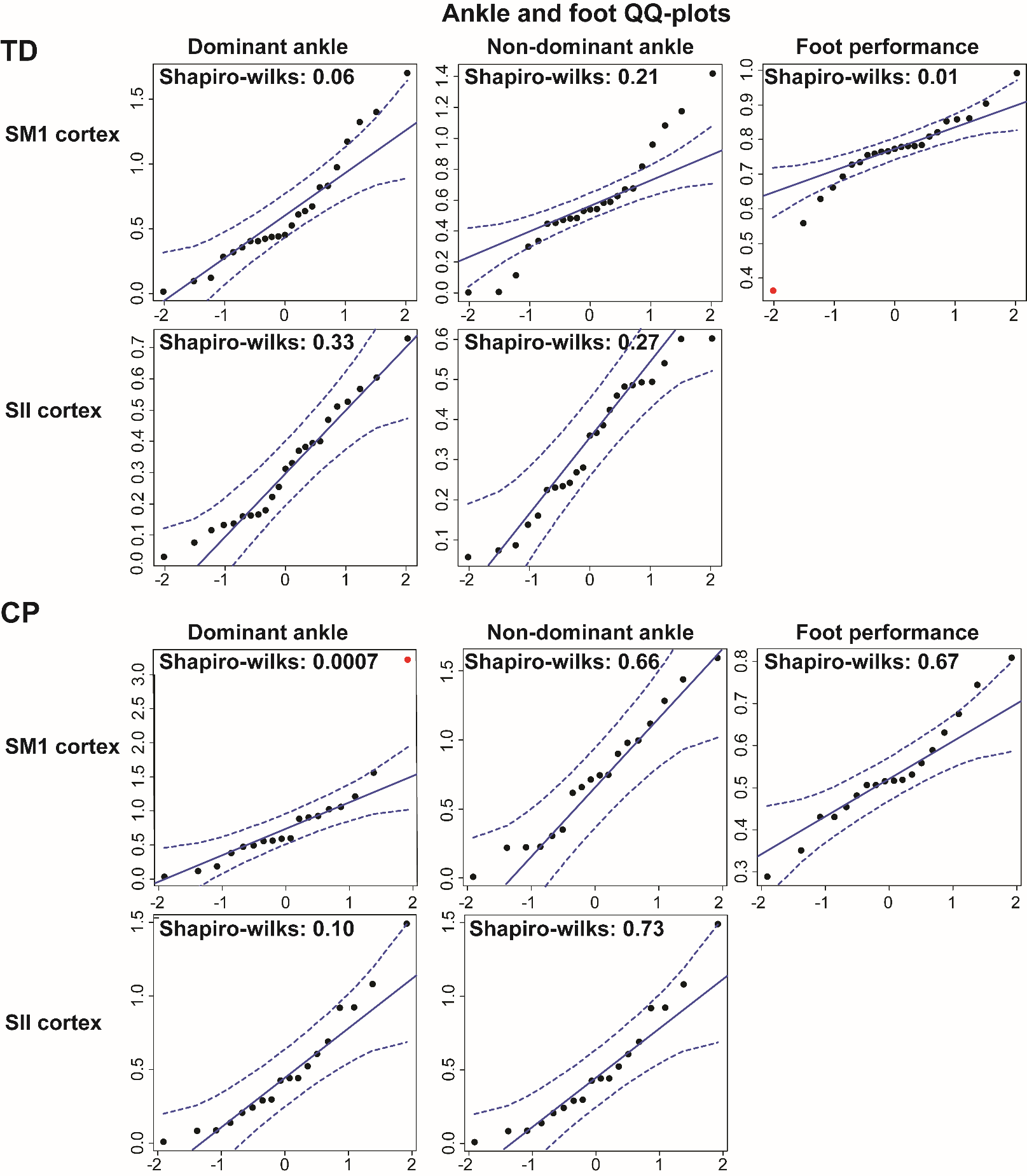


**Figure 2. QQ-plots of ankle and foot related variables**. Figure 2 shows QQ-plots and Shapiro-Wilks test statistics for testing the normality of variables. Outliers are marked in red.

## Multivariate assumptions

Multivariate normality was tested using Henze-Zirkler test. Absence of multicollinearity among the dependent variables was checked using variance inflation factor (VIF). Box’s M-test (Box 1949) was used to determine if variances are equal. Multivariate outlier analysis was performed by Mahalanobis distance (R package: ‘dprep’, function: ‘mahaout’).

## Multivariate assumption results

Response strength and sensorimotor performance variables followed multivariate normal distribution for both hand and feet tests (Henze-Zirkler test for the hand: p = 0.61 for TD group, p = 0.31 for CP group; Henze-Zirkler test for the feet : p = 0.72 for TD group, p = 0.19). Largest maximum variance inflation factor was 3.71 among the dependent variables indicating that there was no multicolinearity (*i.e.* < 10). Equality of variances was met (p > 0.001; note that small rejection threshold of 0.001 is recommended for this test). For the finger and hand variables, multivariate outlier analysis by Mahalanobis distance revealed three outliers in the TD group and no outliers in the CP group. For the ankle and foot variables, Mahalanobis distance revealed no multivariate outliers in either group.

## Conclusion

Due to different groups sizes, deviations from univariate normality and some outliers, Pillai’s Trace was selected as the test statistic for MANCOVA, being more robust against aforementioned deviations than most other test statistics from the assumptions especially when equality of variances is met (Ateş et. al, 2019, Olson 1974, Pillai 1955).

## References

Ateş C., Kaymaz Ö ,2 Kale H. E and Tekindal M. A. Comparison of Test Statistics of Nonnormal and Unbalanced Samples for Multivariate Analysis of Variance in terms of Type-I Error Rates (2019). Computational and Mathematical Methods in Medicine . 2019.

Box GEP. 1949. A general distribution theory for a class of likelihood criteria. Biometrika. 36:317–346.

Olson C. L. Comparative Robustness of Six Tests in Multivariate Analysis of Variance *(*1974). Journal of the American Statistical Association. 69 (348) 894-908.

Pillai KCS. 1955. Some New Test Criteria in Multivariate Analysis. Ann Math Stat. 26:117–121.
