# Supplementary material for: Stronger proprioceptive BOLD-responses in the somatosensory cortices reflect worse sensorimotor function in adolescents with and without cerebral palsy
Source: Neuroimage Clin. 2021 Aug 21;32:102795. doi: 10.1016/j.nicl.2021.102795 (PMC8411230; doi:10.1016/j.nicl.2021.102795)
Supplement: Supplementary data 2 [file mmc2.docx]

**Table 1. AAL region of finger stimulation ROIs.**

|  | **Dominant** | | |  |  | **Non-Dominant** | |
| --- | --- | --- | --- | --- | --- | --- | --- |
|  | **SM1** | | **SII** |  | **SM1** | | **SII** |
| **Participant** | | | |  |  | | |
| 1 | Postcentral_L | | SupraMarginal_L | | Postcentral_R | | Rolandic_Oper_R |
| 2 | Parietal_Inf_L | | SupraMarginal_L | | Precentral_R | | SupraMarginal_R |
| 3 | Precentral_L | | Postcentral_L | | Postcentral_R | | Rolandic_Oper_R |
| 4 | Postcentral_L | | Postcentral_L | | Postcentral_R | | SupraMarginal_R |
| 5 | Precentral_L | | Temporal_Sup_L | | Postcentral_R | | Rolandic_Oper_R |
| 6 | **undefined** | | Temporal_Sup_L | | Postcentral_R | | Rolandic_Oper_R |
| 7 | Postcentral_L | | SupraMarginal_L | | Postcentral_R | | Rolandic_Oper_R |
| 8 | **undefined** | | Rolandic_Oper_L | | Precentral_R | | Rolandic_Oper_R |
| 9 | Postcentral_L | | Temporal_Sup_L | | Postcentral_R | | SupraMarginal_R |
| 10 | Postcentral_R | | Rolandic_Oper_R | | **undefined** | | **undefined** |
| 11 | **undefined** | | Postcentral_L | | Postcentral_R | | Rolandic_Oper_R |
| 12 | Postcentral_L | | Rolandic_Oper_L | | Precentral_R | | Rolandic_Oper_R |
| 13 | Postcentral_L | | SupraMarginal_L | | Postcentral_R | | Rolandic_Oper_R |
| 14 | Postcentral_L | | Rolandic_Oper_L | | Postcentral_R | | Temporal_Sup_R |
| 15 | Postcentral_L | | Temporal_Sup_L | | Postcentral_R | | Rolandic_Oper_R |
| 16 | Postcentral_L | | SupraMarginal_L | | Postcentral_R | | Rolandic_Oper_R |
| 17 | Postcentral_L | | Postcentral_L | | Precentral_R | | Rolandic_Oper_R |
| 18 | Postcentral_R | | SupraMarginal_R | | Postcentral_L | | Postcentral_L |
| 19 | Postcentral_L | | Rolandic_Oper_L | | Precentral_R | | Rolandic_Oper_R |
| 20 | **undefined** | | Postcentral_L | | Postcentral_R | | Rolandic_Oper_R |
| 21 | Postcentral_L | | Rolandic_Oper_L | | Postcentral_R | | Rolandic_Oper_R |
| 22 | Postcentral_L | | SupraMarginal_L | | Postcentral_R | | Rolandic_Oper_R |
| 23 | Postcentral_L | | SupraMarginal_L | | Postcentral_R | | Rolandic_Oper_R |
|  | ROIs: Undefined / outside cortex: 4**/2** | | ROIs: Undefined / outside cortex: 0**/0** | | ROIs: Undefined / outside cortex: 1**/1** | | ROIs: Undefined / outside cortex: 1**/0** |
| **CP** | |  |  |  |  |  |  |
| 24 | Postcentral_R | | Rolandic_Oper_R | | Postcentral_L | | Insula_L |
| 25 | Postcentral_R | | Rolandic_Oper_R | | None* | | None* |
| 26 | Postcentral_R | | Rolandic_Oper_R | | **undefined** | | Temporal_Inf_L |
| 27 | **undefined** | | Rolandic_Oper_R | | Postcentral_L | | SupraMarginal_L |
| 28 | Postcentral_L | | Rolandic_Oper_L | | Frontal_Sup_R | | Postcentral_R |
| 29 | Postcentral_R | | Rolandic_Oper_R | | Postcentral_L | | **undefined** |
| 30 | Postcentral_L | | SupraMarginal_L | | Postcentral_R | | Rolandic_Oper_R |
| 31 | Postcentral_R | | SupraMarginal_R | | Postcentral_L | | Postcentral_L |
| 32 | Precentral_R | | Temporal_Sup_R | | Postcentral_L | | SupraMarginal_L |
| 33 | Postcentral_L | | Postcentral_L | | Frontal_Sup_R | | Temporal_Sup_R |
| 34 | **undefined** | | Rolandic_Oper_R | | **undefined** | | Rolandic_Oper_L |
| 35 | **undefined** | | SupraMarginal_R | | **undefined** | | Postcentral_L |
| 36 | Postcentral_L | | Temporal_Sup_L | | Postcentral_R | | Postcentral_R |
| 37 | Postcentral_R | | Rolandic_Oper_R | | Precentral_L | | SupraMarginal_L |
| 38 | Precentral_R | | Rolandic_Oper_R | | Postcentral_L | | SupraMarginal_L |
| 39 | Postcentral_L | | SupraMarginal_L | | **undefined** | | Heschl_R |
| 40 | Postcentral_L | | SupraMarginal_L | | Precentral_R | | Rolandic_Oper_R |
| 41 | Postcentral_L | | Rolandic_Oper_L | | Postcentral_R | | Rolandic_Oper_R |
|  | ROIs: Undefined / outside cortex:3**/**0 | | ROIs: Undefined / outside cortex: 0**/**0 | | ROIs: Undefined / outside cortex: 4**/1** | | ROIs: Undefined / outside cortex: 1**/**0 |
|  |  |  |  |  |  |  |  |

Table shows regions-of-interest of regions according to the AAL labeling. The table also shows the number of undefined ROIs and ROIs that were located outside the MNI152 template brain. *We were unable to get this participant’s hand to the movement actuator, values imputed for response strength of the non-dominant hand.

**Table 2. AAL region of ankle stimulation ROIs.**

|  | **Dominant** | | | |  | **Non-Dominant** | | |
| --- | --- | --- | --- | --- | --- | --- | --- | --- |
|  | **SM1** | | **SII** | | **SM1** | | **SII** | |
| **Participant** | |  |  |  |  |  |  |  |
| 1 | Paracentral_Lobule_L | | | Temporal_Sup_L | Paracentral_Lobule_R | | | SupraMarginal_R |
| 2 | Paracentral_Lobule_L | | | SupraMarginal_L | Paracentral_Lobule_R | | | **undefined** |
| 3 | Precuneus_L | | | SupraMarginal_L | Parietal_Sup_R | | | Temporal_Sup_R |
| 4 | Paracentral_Lobule_L | | | Rolandic_Oper_L | Postcentral_R | | | **undefined** |
| 5 | Postcentral_L | | | SupraMarginal_L | Postcentral_R | | | Rolandic_Oper_R |
| 6 | Paracentral_Lobule_L | | | SupraMarginal_L | Paracentral_Lobule_R | | | Rolandic_Oper_R |
| 7 | Precuneus_L | | | SupraMarginal_L | Paracentral_Lobule_R | | | SupraMarginal_R |
| 8 | **undefined** | | | **undefined** | Paracentral_Lobule_L | | | SupraMarginal_L |
| 9 | Paracentral_Lobule_L | | | SupraMarginal_L | Postcentral_R | | | SupraMarginal_R |
| 10 | Paracentral_Lobule_R | | | **undefined** | Paracentral_Lobule_L | | | Rolandic_Oper_L |
| 11 | Paracentral_Lobule_L | | | SupraMarginal_L | Postcentral_R | | | Rolandic_Oper_R |
| 12 | Paracentral_Lobule_L | | | Temporal_Sup_L | Supp_Motor_Area_R | | | **undefined** |
| 13 | Precuneus_L | | | Temporal_Sup_L | Paracentral_Lobule_R | | | **undefined** |
| 14 | Postcentral_L | | | Temporal_Sup_L | Postcentral_R | | | SupraMarginal_R |
| 15 | Parietal_Sup_L | | | SupraMarginal_L | Paracentral_Lobule_R | | | Rolandic_Oper_R |
| 16 | Paracentral_Lobule_L | | | Temporal_Sup_L | Postcentral_R | | | Rolandic_Oper_R |
| 17 | Paracentral_Lobule_L | | | Rolandic_Oper_L | Paracentral_Lobule_R | | | Rolandic_Oper_R |
| 18 | Postcentral_R | | | Rolandic_Oper_R | Paracentral_Lobule_L | | | Rolandic_Oper_L |
| 19 | Paracentral_Lobule_L | | | SupraMarginal_L | Postcentral_R | | | Rolandic_Oper_R |
| 20 | Precuneus_L | | | **undefined** | Postcentral_R | | | SupraMarginal_R |
| 21 | Precuneus_L | | | Rolandic_Oper_L | Paracentral_Lobule_R | | | **undefined** |
| 22 | Postcentral_L | | | SupraMarginal_L | Paracentral_Lobule_R | | | Rolandic_Oper_R |
| 23 | Parietal_Sup_L | | | **undefined** | Postcentral_R | | | SupraMarginal_R |
|  | ROIs: Undefined / outside cortex: 1/0 | | | ROIs: Undefined / outside cortex: 4**/**0 | ROIs: Undefined / outside cortex: 0**/**0 | | | ROIs: Undefined / outside cortex: 5**/**0 |
| **HP** | |  |  |  |  |  |  |  |
| 24 | Paracentral_Lobule_R | | | Rolandic_Oper_R | Paracentral_Lobule_L | | | SupraMarginal_L |
| 25 | Postcentral_R | | | SupraMarginal_R | Paracentral_Lobule_L | | | Temporal_Mid_L |
| 26 | Paracentral_Lobule_R | | | Temporal_Sup_R | Precuneus_L | | | Temporal_Mid_L |
| 27 | **undefined** | | | **undefined** | Paracentral_Lobule_L | | | SupraMarginal_L |
| 28 | Supp_Motor_Area_L | | | SupraMarginal_L | Paracentral_Lobule_R | | | Rolandic_Oper_R |
| 29 | Postcentral_R | | | Rolandic_Oper_R | Paracentral_Lobule_L | | | **undefined** |
| 30 | Precuneus_L | | | Temporal_Sup_L | Paracentral_Lobule_R | | | Temporal_Sup_R |
| 31 | Paracentral_Lobule_R | | | SupraMarginal_R | Paracentral_Lobule_L | | | Temporal_Sup_L |
| 32 | Paracentral_Lobule_R | | | Temporal_Sup_R | Paracentral_Lobule_L | | | Temporal_Sup_L |
| 33 | Paracentral_Lobule_L | | | Rolandic_Oper_L | Precentral_R | | | **undefined** |
| 34 | Supp_Motor_Area_R | | | Rolandic_Oper_R | Precuneus_L | | | Insula_L |
| 35 | Paracentral_Lobule_L | | | SupraMarginal_L | Paracentral_Lobule_R | | | Rolandic_Oper_R |
| 36 | Paracentral_Lobule_L | | | SupraMarginal_L | Postcentral_R | | | SupraMarginal_R |
| 37 | Paracentral_Lobule_R | | | Rolandic_Oper_R | Paracentral_Lobule_L | | | SupraMarginal_L |
| 38 | Paracentral_Lobule_R | | | SupraMarginal_R | Paracentral_Lobule_L | | | Insula_L |
| 39 | Precuneus_L | | | Rolandic_Oper_L | Postcentral_R | | | **undefined** |
| 40 | Precuneus_L | | | SupraMarginal_L | Postcentral_R | | | SupraMarginal_R |
| 41 | Precuneus_L | | | SupraMarginal_L | Postcentral_R | | | SupraMarginal_R |
|  | ROIs: Undefined / outside cortex: 1/0 | | | ROIs: Undefined / outside cortex: 1**/1** | ROIs: Undefined / outside cortex: 0**/0** | | | ROIs: Undefined / outside cortex: 0**/0** |

Table shows regions-of-interest of regions according to the AAL labeling
